# Genome Sequence of Organophosphorus Pesticide-Degrading Bacterium Pseudomonas stutzeri Strain YC-YH1

**DOI:** 10.1128/genomeA.00192-15

**Published:** 2015-03-26

**Authors:** Yan-hua Shi, Lei Ren, Yang Jia, Yan-chun Yan

**Affiliations:** Graduate School of Chinese Academy of Agricultural Sciences, Beijing, China

## Abstract

Pseudomonas stutzeri strain YC-YH1, isolated from pesticide-polluted soil, efficiently degrades organophosphorus pesticides (OPPs) such as chlorpyrifos, parathion-methyl, triazophos, and parathion. Here, we report the genome sequence (4.83 Mb) of P. stutzeri YC-YH1 to facilitate further investigation of the OPP-degrading mechanism.

## GENOME ANNOUNCEMENT

Biodegradation is generally recognized as the mass balance, the most important route of pesticide degradation (1). Organophosphorus pesticides (OPPs) account for ~38% of the world’s total pesticide usage, which includes more than 100 OPPs that are in use, such as chlorpyrifos, parathion-methyl, triazophos, malathion, and phosphamidon (2). Genetic and biochemical studies of OPP degradation were greatly promoted by microbial genome sequencing in the past and recently. Several genomes of OPP-degrading strains have been published to date (3–5), including Pseudomonas spp., Burkholderia spp., Burkholderia zhejiangensis, etc. Strain YC-YH1 was isolated from pesticide-contaminated soil from Xingtai, Hebei Province, China. Strain YC-YH1 efficiently degrades several OPPs, such as chlorpyrifos, parathion-methyl, triazophos, and parathion. Strain YC-YH1 was identified as Pseudomonas stutzeri using 16S rRNA sequence analysis and a Biolog GEN III MicroStation bacterial identification system. The genome sequence of P. stutzeri strain YC-YH1 may improve our understanding of the strain’s high ability and genetic information for biodegradation of OPPs. Here, we present a summary, classification, and set of features for P. stutzeri strain YC-YH1 together with a description of the genomic sequencing and annotation.

The genome sequence of P. stutzeri strain YC-YH1 was obtained using Illumina High-Seq 2000 sequencing with a 500-bp paired-end library and a 3-kb mate-pair library. A total of 2,405,832,532 bp of original data and 19,584,632 high-quality reads were generated (approximately 481× coverage of the genome), with an average G+C content of 64%. The reads were assembled using SOAPdenovo (http://soap.genomics.org.cn/soapdenovo.html, version 2.04) (6). The genome of strain YC-YH1 has 56 scaffolds, with a calculated 5,079,790-bp total length and *N*_50_ contig size of 467,282 bp. Tandem repeats were identified by Tandem Repeat Finder (http://tandem.bu.edu/trf/trf.html, version4.02) (7). Annotation was performed by the NCBI Prokaryotic Genomes Automatic Annotation Pipeline (PGAAP) (http://www.ncbi.nlm.nih.gov/genome/annotation_prok/). The genome sequence contains 4,253 candidate protein-coding sequences (CDSs), 1 23S rRNA gene, 1 noncoding RNA gene, and 52 tRNA genes. The CDSs were searched against the KEGG and Clusters of Orthologous Groups (COG) databases to analyze the gene functions and metabolic pathways (8). In all, 3,554 proteins were assigned to COG families and 735 CDSs were included in 91 pathways.

The analysis of the genome data from P. stutzeri YC-YH1 revealed that there was a gene of methyl parathion hydrolase (*mpd*, EF515812) and a gene of organophosphorus hydrolase (*ophC2*, EU651813), which contributed to the degradation of most OPPs. As to the further degrading related genes and regulation mechanism, it is still unknown and further investigation is needed. The genome information and annotation of P. stutzeri YC-YH1 is valuable for future research to investigate OPP degradation in the environment and to provide the possibility for bioremediation of OPP-contaminated soil and water.

### Nucleotide sequence accession number.

The complete genome sequence of P. stutzeri YC-YH1 was deposited at GenBank under the accession no. JUDR00000000.
